# Editorial: Manual Skills, Handedness, and the Organization of Language in the Brain

**DOI:** 10.3389/fpsyg.2019.00930

**Published:** 2019-04-26

**Authors:** Gregory Króliczak, Claudia L. R. Gonzalez, David P. Carey

**Affiliations:** ^1^Action and Cognition Laboratory, Department of Social Sciences, Institute of Psychology, Adam Mickiewicz University in Poznan, Poznan, Poland; ^2^Department of Kinesiology, University of Lethbridge, Lethbridge, AB, Canada; ^3^Department of Neuroscience, University of Lethbridge, Lethbridge, AB, Canada; ^4^Perception, Action and Memory Research Group, School of Psychology, Bangor University, Bangor, United Kingdom

**Keywords:** hand preference, cerebral dominance, brain functioning, sensorimotor control, higher-order processing, skilled actions, praxis

Hand preference and cerebral dominance for some aspects of language processing are hallmarks of human brain functioning. Yet, their mutual relationships, similar to interrelations between hemispheric dominance for low-level sensorimotor control of the hand and the representations of higher-order, skilled actions (praxis) still remain unclear. Whereas in some accounts (Liepmann, 1900, 1908; Geschwind and Galaburda, 1985; Heilman, 1997; see also Goldenberg, 2013b) right handedness reflects (at least in part) the functioning of the left-lateralized manual praxis system, evidence from majority of left-handers weakens such a notion because they often represent praxis skills in their motor non-dominant—left—hemispheres, too (Lausberg et al., 1999; Frey et al., 2005; Goldenberg, 2013a; see also Gonzalez and Goodale, 2009; Grabowska et al., 2012; Haberling and Corballis, 2015; Kroliczak et al., 2016; cf. Carey et al., 2015). Although the putative links between praxis and language, and their interactions with handedness, have been long considered (Dejerine and Andre-Thomas, 1912; Heilman et al., 1973, 1974; McManus, 1985; Annett and Alexander, 1996; Meador et al., 1999), more recent studies clarify their relationships (Króliczak et al., 2011; Vingerhoets et al., 2013; Biduła and Króliczak, 2015; Goldenberg and Randerath, 2015; cf. Goldenberg, 2013b), further strengthening the idea that they are contingent on each other (Vingerhoets, 2014; Króliczak et al., 2018). Moreover, evidence from individuals with rarer forms of brain dominance now supports the idea that there is a longstanding evolutionary origin to the cerebral arrangement and distribution of both related and complementary skills, e.g., praxis and language vs. attention (Grabowska et al., 1994; Corballis, 2003; Cai et al., 2013; Goldenberg, 2013b).

The primary goal of this Research Topic is to present new pieces of evidence on the neural and functional organization of language and praxis, their links (or lack of thereof) with handedness and low-level motor skills, as well as behavioral consequences of their representations for other functions. Among the 12 contributing Original Research Articles, the considered functions include short-term tactile learning of Braille reading, visual word and number processing, and visuospatial discrimination. Yet, because the neural underpinnings of these functions are often strongly lateralized in the human brain, and may have common ancestry, their evolution and development is discussed in two Hypothesis and Theory articles.

## Evolution and Early Development of Cerebral and Behavioral Asymmetries

The evolution of language and tool manufacture is considered by Corballis in the context of behavioral asymmetries that emerged in humans. Evidence is discussed that such asymmetries must have developed in an independent manner, triggered by multi-genetic sources, rather than a single overriding principle. It is also emphasized that representations of language and tool-use skills are rather poorly correlated with handedness. The relation of the individual development of hand preference to the critical development of human basic sensorimotor and cognitive abilities is, nevertheless, assumed in Michel. Based on earlier ideas that hand preference acquisition precedes unimanual object manipulation, and that both these skills must precede role-differentiated bimanual manipulation of objects, this contribution provides a description of an ideal paradigm for testing their development and relationships. The importance of studying developmental differences of cognitive skills across handedness is also emphasized.

## Language Laterality, its Sources, and (in)Activity-Dependent Word Processing and Learning

Unique characteristics of atypical organization of language are considered by Biduła et al. Whereas, most of atypical cases are indeed found in left-handers, they are also present in ambidextrous and right-handed people (cf. Carey and Johnstone, 2014). Indeed, Biduła et al. demonstrated that although group results indicate mirror-reversed organization of language in atypical participants, evidence for this is less compelling at an individual level of analysis. The relationships between language laterality and handedness are also discussed by Schmitz et al., but now from the point of genetic influences. Evidence is shown that handedness and language organization are complex phenotypes that are ontogenetically independent. This report ends with conclusions that genes involved in ontogenesis of handedness contribute primarily to structural development, whereas genes underlying language laterality also contribute to the development of other cognitive processes (but seem also associated with mental and neurological disorders).

Given language-praxis links, certain kinds of actions, or inaction, could affect language processing and learning. For example, changes in motor system functioning could flexibly influence comprehension and acquisition of words (cf. Shebani and Pülvermuller, 2018). An intriguing paper by Yasuda et al. demonstrates that while peripheral body states influence action verb processing, in contrast to a strong embodiment view, constrained arm posture affected responses to both manual and non-manual action verbs. The opposite issue, that is, an impact of word processing on movement kinematics was investigated by Rugani et al. They showed that automatic numerical processing affects action execution in a context of kicking small balls with the index finger. Their participants responded faster to small numbers while kicking the ball to the left, and vice versa. Notably, Rugani et al. argue that similar paradigms could be used to study the impact of cognition on action in an unbiased way.

Learning new vocabulary can be a challenge, especially in elder people. Yet, as Heim and collaborators show (Heim et al.), a nap, in contrast to activity or even rest, helps to consolidate language learning. While these results are less relevant to the language-praxis debate, their translation to clinical settings for improvement of speech-language therapy following brain injuries would be welcome. Still, in some circumstances learning to read new words is not possible without the involvement of certain kinds of actions, as in tactile learning of Braille (Debowska et al.). This study established that even short-term tactile training can introduce functional and structural changes in the fusiform gyrus, linked to visual processing of language, including single word reading. This is yet another demonstration how language and praxis can be related.

## When Handedness Does Not Matter, Does it?

Some manual actions seem so simple that one would expect mainly contralateral control of their performance. Yet, as Begliomini et al. show, grasping with the left (dominant) hand in left handers is not controlled only by the right (contralateral) hemisphere. They found increased connectivity with the left hemisphere parieto-frontal resources. Notably, the right (non-dominant) hand is controlled as in right-handers. These outcomes are consistent with a notion that hemispheric specialization for higher-order visuomotor control does not depend on handedness (Gonzalez et al., 2006). Nevertheless, reports on the impact of handedness, the used hand/eye, and/or other cognitive abilities on performance of the line bisection task (Ochando and Zago), and left-right discrimination (Constant and Mellet) reveal a more complex picture. In the line bisection task, performance depends on integration of differently weighted visuospatial hemispheric mechanisms, the motor component of the used hand, and individual laterality factors. When they are congruent, the strongest behavioral biases are observed. As to left-right discrimination, left-handers were found better at identifying their left hands and verifying “left” propositions. Nonetheless, numerous interactions of other factors provide new insights into the links between cognitive skills and left-right discrimination.

## Functions Still To-be-Tested in Left-Handers

The last three papers focus entirely on specific aspects of motor control. They shed new light on the impact of spatial alignment and response hand in processing visual illusions (Scocchia et al.), competition between functional and situational affordances (Roche and Chainay), and the influence of action mode on efficiency in rule- vs. plan-based movements (Scheib et al.). Of course, the studied skills are less likely to depend on linguistic representations. Yet, although some differences contingent on the responding hand were suggested, they are less likely to emerge when directed at tools. As such, these approaches can stimulate new research and reveal new findings of theoretical interest for our debate.

## Conclusions

This Research Topic highlights the findings on the relationships between manual skills and language, and their putative links to handedness and associated motor functions. Research showing both similarities and disparities in their organization in right-handed and left-handed (but also ambidextrous) individuals is featured. The debate includes the evolution and early development of cerebral and behavioral asymmetries, as well as their genetic foundations. We hope that further discussions and research ideas will emerge out of this work.

## Author Contributions

GK conceived this work and drafted the editorial. CG and DC contributed intellectually to this work, revised, and approved the draft for publication.

### Conflict of Interest Statement

The authors declare that the research was conducted in the absence of any commercial or financial relationships that could be construed as a potential conflict of interest.
